# Endogenous Cholinergic Inputs and Local Circuit Mechanisms Govern the Phasic Mesolimbic Dopamine Response to Nicotine

**DOI:** 10.1371/journal.pcbi.1003183

**Published:** 2013-08-15

**Authors:** Michael Graupner, Reinoud Maex, Boris Gutkin

**Affiliations:** 1 Group for Neural Theory, Laboratoire de Neurosciences Cognitives, INSERM Unité 969, Départment d'Etudes Cognitives, École Normale Supérieure, Paris, France; 2 Center for Neural Science, New York University, New York, New York, United States of America; 3 CNRS, Paris, France; University College London, United Kingdom

## Abstract

Nicotine exerts its reinforcing action by stimulating nicotinic acetylcholine receptors (nAChRs) and boosting dopamine (DA) output from the ventral tegmental area (VTA). Recent data have led to a debate about the principal pathway of nicotine action: direct stimulation of the DAergic cells through nAChR activation, or disinhibition mediated through desensitization of nAChRs on GABAergic interneurons. We use a computational model of the VTA circuitry and nAChR function to shed light on this issue. Our model illustrates that the α4β2-containing nAChRs either on DA or GABA cells can mediate the acute effects of nicotine. We account for *in vitro* as well as *in vivo* data, and predict the conditions necessary for either direct stimulation or disinhibition to be at the origin of DA activity increases. We propose key experiments to disentangle the contribution of both mechanisms. We show that the rate of endogenous acetylcholine input crucially determines the evoked DA response for both mechanisms. Together our results delineate the mechanisms by which the VTA mediates the acute rewarding properties of nicotine and suggest an acetylcholine dependence hypothesis for nicotine reinforcement.

## Author Summary

Nicotine is the major addictive substance in tobacco smoke. Nicotine exerts its control over neural circuits through nicotinic acetylcholine receptors that normally respond to endogenous acetylcholine. Activation of dopamine neurons in the mesolimbic dopaminergic circuits, which signal motivational properties of actions and stimuli, is at the heart of mediating nicotine reward and dependence. However, major questions have remained unsettled over the precise mechanisms by which nicotine usurps dopaminergic signaling: through receptor activation on dopamine neurons or through receptor desensitization on local inhibitory interneurons. Here we reconcile this debate by showing that both mechanisms are possible. Most notably we present a novel hypothesis suggesting that the mechanisms for nicotine action are state-dependent; they are controlled by the rate of the endogenous cholinergic input to the dopaminergic circuits.

## Introduction

The ventral tegmental area (VTA) is a key dopaminergic structure for signaling reward and motivation as well as for the acquisition of drug-reinforced behavior [1], [2]. Nicotine (Nic) stimulates nicotinic acetylcholine receptors (nAChRs) in the VTA [3] boosting dopamine (DA) output to its targets such as the nucleus accumbens [4] and thereby playing a cititcal role in the mediation of nicotine reward and dependence [5]–[7]. Yet, despite a wealth of data on the outcome of nicotine action, the precise mechanisms by which nicotine usurps control over DA signaling remain debated.

Release of endogenous acetylcholine (ACh) from cholinergic projections [8] causes activation of nAChRs in the VTA [9]. The rapid breakdown of ACh by acetylcholinesterase precludes significant nAChR desensitization [9], [10]. Exogenous nicotine is not hydrolyzed [11] and thus activates and subsequently desensitizes nAChRs within seconds to minutes [10], [12]. The various subtypes of nAChRs exhibit markedly different activation/desensitization kinetics and distinct affinities for ACh and Nic, as well as different expression targets [7], [13]. The low-affinity α7 subunit-containing nAChRs desensitize rapidly (∼ms) [14] and are found in the VTA presynaptically on the glutamatergic terminals [15]. The high-affinity α4β2 subunit-containing nAChRs desensitize relatively slowly (∼sec) and are located postsynaptically on the DA and GABAergic cells [13]. Studies on knockout mice suggest that the α4β2 nAChRs mediate most of the Nic-evoked currents and the acute reinforcing effects of nicotine [16], while α7 nAChRs appear to contribute to the fine-tuning of the DA response to nicotine [17].

A major outstanding question is whether nicotine acts directly on the DA neurons through the activation of α4β2 nAChRs or affects the local GABA interneurons and thereby the inhibition of DA neurons. *In vitro* data suggest that the increased activity of DA neurons is due to nicotine desensitizing the α4β2 nAChRs, decreasing the endogenous cholinergic drive to GABA neurons and resulting in disinhibition of DA cells [18], [19]. *In vivo* studies emphasize the role of nicotine-evoked direct activation of β2-containing nAChRs expressed on the DA neurons [17].

We set out to clarify the mechanisms linking the nicotine-triggered nAChR activation/desensitization and nicotine control of DA signaling. In order to do so we built a neuronal network model that includes the afferent and local VTA connectivity as well as the location and activation/desensitization properties of the different nAChR subtypes. Our results show that each of the two mechanisms, direct excitation and disinhibition, requires distinct conditions for afferent input strengths and cellular properties in order to account for the nicotine-evoked DA boost. We develop a series of experimental protocols to disambiguate the disinhibition vs. the direct stimulation pathways and reveal that the endogenous cholinergic input rate dictates the DA response to nicotine for both.

## Results

**Figure 1:**
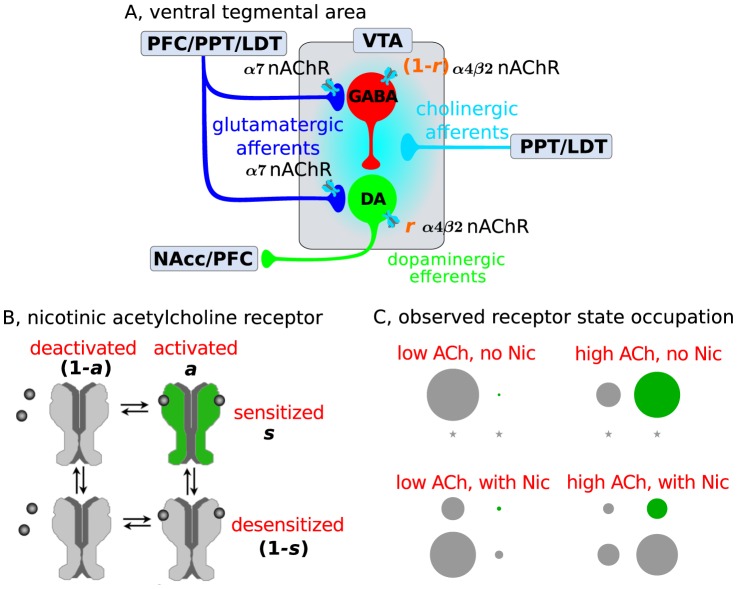
Scheme of the ventral tegmental area and the states of nicotinic acetylcholine receptors. (A) Afferent inputs and circuitry of the ventral tegmental area (VTA). The GABAergic neuron population (red) inhibits locally the dopaminergic neuron population (DA, green) [40], [43], [44]. This local circuit receives excitatory glutamatergic input (blue lines) from the prefrontal cortex (PFC) [68]–[71], the laterodorsal tegmental nucleus (LDT) and the pedunculopontine tegmental nucleus (PPT) [72]–[74]. The LDT and the PPT furthermore furnish cholinergic projections (cyan lines) to the VTA [8]. nAChRs are found at presynaptic terminals of glutamatergic projections (α7-containing receptors), on GABAergic neurons (α4β2 nAChRs) and DA neurons (α4β2 nAChRs). *r* is a parameter introduced in the model to change continuously the dominant site of α4β2 nAChR action. Dopaminergic efferents (green) project, amongst others, to the nucleus accumbens and the PFC. (B) Two-gate model of nicotinic acetylcholine receptors. Activation (horizontal) and desensitization (vertical) of nAChRs are two independent transitions in the model, *i.e*, the receptor can exist in four different states: (i) deactivated/sensitized (up-left), (ii) activated/sensitized (up-right), (iii) deactivated/desensitized (down-left), and (iv) activated/desensitized (down-right). Activation is driven by Nic and ACh and induces a transition from the deactivated/sensitized to the activated/sensitized state (green), the only open state in which the receptor mediates an excitatory current. Desensitization is driven by Nic and ACh if 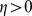. *a* and *s* characterize the fraction of nAChRs in the activated and the sensitized state, respectively (modified from [22]). (C) α4β2 nAChR state occupation as described by the model for different Nic and ACh concentrations (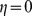). The area of the circles represents the fraction of receptors in each of the four states (alignment as in panel B). The occupation of receptor states is shown for long-term exposures to low (0.1 µM) and high (100 µM) ACh, without and with 1 µM nicotine. A star means that the respective state is not occupied.

Our minimal local circuit model of the VTA reflects the glutamatergic (Glu) and cholinergic (ACh) afferents to the DA and GABA cells in the VTA, as well as local inhibition of DA cells by GABA neurons (see Fig. 1A). Importantly we explicitly model the subtype-specific activation and desensitization of α4β2 and α7 nAChRs since these subtypes have been shown to be predominant in mediating nicotine effects in the VTA [13], [16], [17]. Further evidence supports the critical role of α4-containing nAChRs for Nic action in the VTA [20], [21]. Based on available data we model the α7 nAChRs as placed at presynaptic Glu terminals where they affect Glu input strength [15]. We model the α4β2 nAChR as placed somatically on both the DA and the GABA neurons. The relative α4β2 nAChR expression level (DA/GABA proportional density) is controlled in our model by a fraction parameter *r* which allows us to shift continuously the balance of α4β2 nAChR-mediated effects from GABA cells (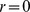) to DA cells (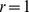; Fig. 1A). Overall, the model augments the mean-field firing-rate description of the relevant neuronal populations with subtype specific receptor currents in order to study neuronal activity in response to endogenous (ACh) and exogenous (Nic) ligands acting on nAChRs (see Models).

### Kinetics of the subunit-specific nAChR model in response to Nic and/or Ach

Since the nAChRs are the exclusive points of action for Nic that affect DA activity in the VTA, a model of the nAChR that incorporates the response properties of the considered receptor subtypes is key for our approach. In addition, since the circuit model describes the mean activities of the DA and GABA neuronal populations, we describe the average macroscopic receptor-mediated currents as opposed to the fine details of single receptor kinetics and pharmacology. Accordingly, the receptor activation is elicited by the mean input rate of endogenous ACh release which is expressed as a concentration. We use a classical 4-state model of the nAChR adapted from Katz and Thesleff [22], [23] (see Text S2.) briefly described in [24]) accounting for subtype-specific activation and desensitization as recorded in response to Nic and ACh [25]–[30].

**Figure 2:**
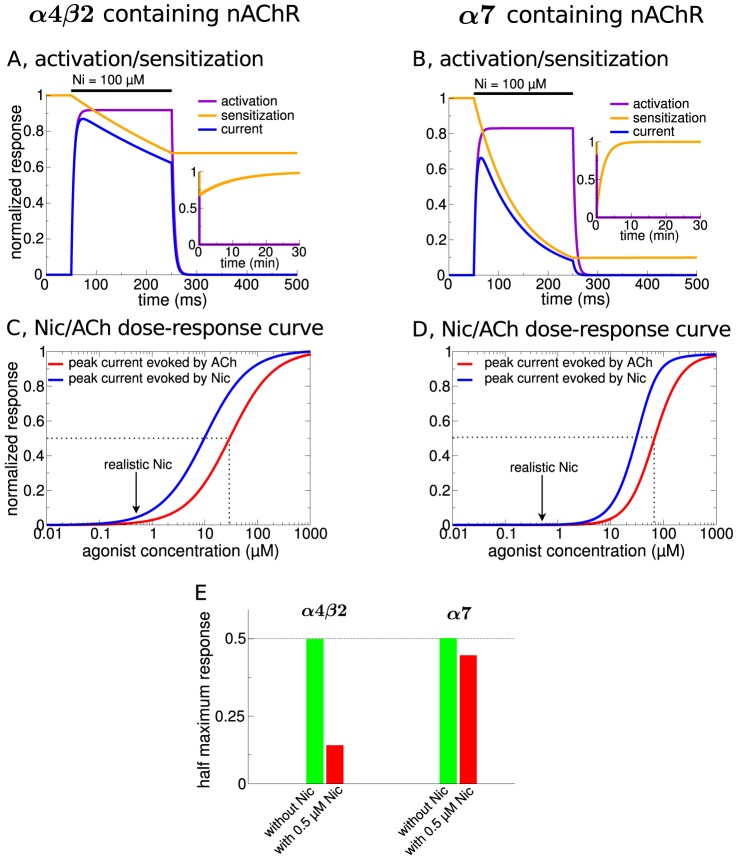
Nicotinic acetylcholine receptor model responses to nicotine and acetylcholine. Response properties of α4β2 (panels A,C,E) and α7 nAChRs (panels B,D,E). (A&B) Dynamics of α4β2- (A) and α7-containing nAChRs (B) in response to Nic. The dynamics of the activation, *a*, (purple lines) and sensitization, *s*, (orange lines) are shown during and after the exposure to a constant Nic concentration of 100 µM for 200 ms starting at 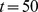 ms. The normalized receptor activation, 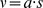, is shown in blue and is proportional to the actual current. The inset shows the dynamics of the same variables on a longer time scale. (C&D) Dose-response curves of α4β2- (C) and α7-containing nAChRs (D) in response to Nic and ACh. The peak current mediated by the receptor during a 200 ms exposure to the respective agonist concentration is shown. The responses to Nic (ACh) are depicted in blue (red). The arrows indicate physiologically relevant nicotine concentrations [11], [31]. The half-maximum effective concentrations for ACh-evoked responses are indicated by the dotted black lines. (E) Reduction of the half-maximum response evoked by ACh in the presence of 0.5 µM Nic. The half-maximum effective concentration of the peak current in the absence of Nic is 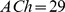 µM for α4β2, and 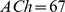 µM for α7 nAChRs (green bars, see C and D). The red bars show the peak current in response to the same ACh concentration in the presence of a constant concentration of 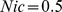 µM. See Table 1 for parameters.

Fast nAChR activation in response to acetylcholine and/or nicotine (transition from deactivated/sensitized to activated/sensitized in the model; see Fig. 1B) gives rise to an initial peak current, and the slower desensitization reduces the current during sustained presence of the agonist (Fig. 2A and B for α4β2 and α7, respectively). After wash-out of agonists, the receptor recovers to the deactivated/sensitized state within seconds-to-minutes [7], [13] (see insets in Fig. 2A and B).

In order to account for the response differences between the two considered nAChR subtypes, two distinct sets of parameters were identified (see Models with respect to details of the parameter adjustment). Most importantly, the potency of nicotine and acetylcholine for the α4β2 nAChR is much higher than for the α7 nAChR (see dose-response curves in Fig. 2C and D). Hence the α4β2 but not the α7 nAChRs shows a partial response at the relatively low, physiologically relevant Nic concentration of 0.5 µM [11], [31] (indicated in Fig. 2C and D).

Both receptor subtypes show distinct temporal desensitization dynamics. Desensitization is relatively slow for α4β2 nAChRs: α4β2 nAChR-mediated currents decrease slowly over the course of agonist exposure (Fig. 2A) and the receptor recovers slowly (∼10 min) from the desensitized state after removal of the agonist (inset in Fig. 2A). On the contrary, at high agonist concentrations fast desensitization of α7-containing receptors completely suppresses the evoked current on the time scale of ∼100 ms (Fig. 2B) and the receptor recovers from desensitization within ∼2 min (inset in Fig. 2B).

The α4β2- and α7-nAChR model responses to ACh show another crucial difference when a physiologically relevant concentration of Nic is present (0.5 µM, Fig. 2E). Pretreatment with Nic reduces the half-maximum response by ∼73% for α4β2 nAChRs, whereas this response decreases only by ∼13% for α7 nAChRs (Fig. 2E). This is due to the lower potency of Nic for desensitizing α7 nAChRs as compared to α4β2 nAChRs, *i.e.*, 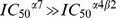 (see Models and Table 1). As a result, a smaller fraction of α7 nAChRs is driven into the desensitized state. Please note that the desensitized fraction of α4β2 nAChRs is ∼70%; their response is not completely abolished at 0.5 µM Nic. While Nic reduces the maximum amplitudes for both receptors, it does not affect the effective half-maximum ACh concentrations [24]. In the absence of ACh, Nic evokes a small activation of the receptors [24] that has also been seen experimentally for α4β2 nAChRs [12].

**Table 1:** Parameters of nAChR activation and desensitization kinetics.

Parameter	Definition	Value	Reference
**α7-containing nAChR**			
*EC*_50_	half-maximum conc. of activation	80 µM	[25], [27], [29], [30]
α	potency of nicotine to evoke response	∼2	[30], [75]
*n_a_*	Hill coefficient of activation	1.73	[25], [29], [30]
*IC*_50_	half-maximum conc. of desensitization by Nic	1.3 µM	[25], [30]
*n_d_*	Hill coefficient of desensitization	2	[25]
*τ*_a_	activation time constant	5 msec	[29]
*K_τ_*	half-maximum conc. of desensitization time constant	1.73 µM	[25]
*n_τ_*	Hill coefficient of des. Time constant	2	[25]
*τ*_max_	maximal des. time constant	2 min	[25]
*τ*_0_	minimal des. time constant	50 msec	this study, [29]
**α4β2-containing nAChR**			
*EC*_50_	half-maximum conc. of activation (ACh)	30 µM	[28]
*α*	potency of Nic to evoke response	∼3	[26], [28]
*n_a_*	Hill coefficient of activation	1.05	[25], [28]
*IC*_50_	half-maximum conc. of desensitization by Nic	0.061 µM	[25]
*n_d_*	Hill coefficient of desensitization	0.5	[25]
*τ*_a_	activation time constant	5 msec	[28]
*K_τ_*	half-maximum conc. of desensitization time constant	0.11 µM	[25]
*n_τ_*	Hill coefficient of des. Time constant	3	[25]
*τ*_max_	maximal des. time constant	10 min	this study
*τ*_0_	minimal des. time constant	500 msec	this study, [28]

In summary, the simple nAChR model is constrained to capture the key properties of the subtype specific responses to Nic and ACh. Most significant for this study is that Nic at physiologically relevant concentrations significantly activates and desensitizes α4β2 but not α7 receptors.

Data used to constrain the model parameters are obtained in the absence of acetylcholinesterase [25]–[30]. Under such conditions, persistent presence of Nic and of ACh both activates *and* desensitizes the nAChRs in the model. However, rapid ACh hydrolysis and a higher efficiency of Nic to desensitize the receptor seem to prevent ACh from desensitizing nAChRs *in vivo* [9], [10], [32]. In the following, we model conditions where acetylcholine is rapidly hydrolyzed and drives only the transition from deactivated to activated state (see Fig. 1B and C), hence desensitization is driven by nicotine only. We also investigate, later in the manuscript, how our results are affected in case of low acetylcholinesterase activity, that is, when also ACh drives receptor desensitization (see “ACh-driven desensitization through low acetylcholinesterase activity”).

### The VTA responses to nicotine *in vitro* and *in vivo*

We next ask whether we can identify the specific circuit-level pathways of nicotine action in the VTA all the while reconciling the *in vitro* and *in vivo* data. First we show that the VTA model endowed with the description of the nAChR dynamics captures *in vitro* nicotine evoked modulation of excitatory and inhibitory inputs to DA neurons [18], [19]. We then demonstrate that a simple change in the constant afferent input strengths allows the model to also account for *in vivo* DA responses in both wild type and nAChR knockout mice [17].

### Nicotine-dependent modulation of excitatory and inhibitory input to DA cells *in vitro*

**Figure 3:**
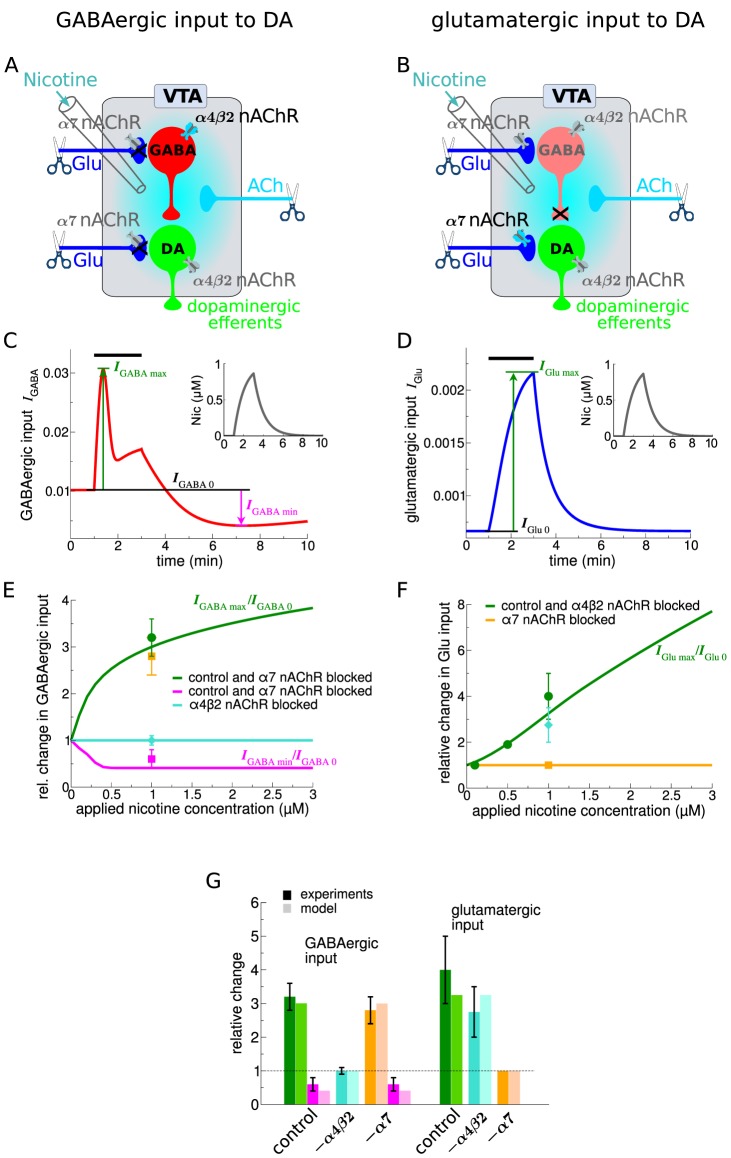
Model VTA responses to nicotine *in vitro*. Left hand panels (A,C,E and left side of panel G) show the results on GABAergic input changes to VTA DA cells, while the panels on the right-hand side (B,D,F and right side of G) depict results on glutamatergic input increases to VTA DA cells in response to 1 µM nicotine for 2 min starting at *t* = 1 min. (A&B) Illustration of the simulated experimental situation during *in vitro* experiments. Grey shaded parts and black crosses show pharmacologically blocked transmission pathways, and the scissors illustrate the truncation of the input pathways *in vitro*. (C&D) Time course of GABAergic, 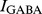, (C) and glutamatergic input, 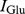, (D) changes to VTA DA neurons during and after Nic exposure (black bar on top of the panels, Nic time-course shown in insets). The increase (green) and the decrease (magenta in C) of the input currents with respect to baseline are illustrated in both panels. (E&F) Maximal change of GABAergic (E) and glutamatergic input currents (F) as a function of the Nic concentration applied. The lines show the results of the model for control conditions (in green in both panels and magenta for decrease in panel E), with α4β2 nAChRs blocked (cyan in panel E, and green in panel F), and with α7 nAChRs blocked (green and magenta in panel E, orange in panel F). The squares show experimental results adapted from [19] (E) and [18] (F) for different experimental situations : control conditions - green squares; with α7 nAChR specific antagonist - orange squares; and with antagonist for non- α7 nAChRs - cyan squares. (G) Comparison of relative input changes between model and experiment for the case of 1 µM nicotine for 2 min. Model results are shown with shaded bars and experimental results with filled bars. Both, GABAergic- (left) and glutamatergic input changes (right) are shown for the three discussed cases : control conditions - green and magenta, α4β2 nAChR blocked - cyan, and α7 nAChR blocked - orange and magenta (experimental data adapted from [18], [19]; 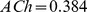 µM and 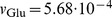 in all panels, see Table 1 and Models section for other parameters).

Recordings from DA neurons in VTA slices show that bath application of 1 µM nicotine initially increases the frequency of IPSCs followed by a drop below baseline after Nic perfusion [19]. Furthermore, 1 µM nicotine evokes a robust enhancement of the spontaneous EPSC frequency in DA neurons [18]. To account for these data we start out by setting the afferent input strength to relatively low levels which characterizes the *in vitro* situation (illustrated by the scissors in Fig. 3A and B).

In line with experiments, the nicotine-evoked initial increase of inhibition and subsequent drop below baseline are exclusively mediated by α4β2 nAChRs (Fig. 3C). The α4β2 nAChRs located on VTA GABAergic cells are activated by application of 1 µM Nic for 2 min. This increases the GABAergic population activity and in turn leads to an increase in the GABA input (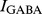) to the DA cells (see green lines in Fig. 3C and E). The first peak of this response arises from fast receptor activation counterbalanced by slower desensitization. The subsequent steady and smaller increase follows the time course of Nic concentration build-up (Fig. 3C). After washout of the drug, a significant fraction of nAChRs remains desensitized and recovers at a slow time-scale (Fig. 3C). This desensitized fraction of nAChRs reduces the GABA cell response to the constant cholinergic input so that 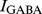 falls below baseline after nicotine is removed (illustrated in magenta in Fig. 3C and E). The recovery time course is governed by the maximal desensitization time constant, 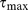, of α4β2 nAChRs. Based on experimental data, that time constant was set to approximately 10 min (see Table 1; [17], [19]). To account for the block of Glu transmission during these experiments we set 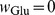 (see Models).

As expected, blocking α4β2 nAChRs in our model abolishes the Nic-evoked modulation in IPSC frequency (cyan line in Fig. 3E, [19]). In contrast, the α7 nAChRs have little or no impact on the response (green line and orange square in Fig. 3E, [19]). Our model accounts for the *in vitro* data in both the control, the α4β2- and the α7-nAChR blocked conditions. Note that in order to do so, the average cholinergic drive to the GABAergic cells needs to be set at 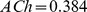 µM, giving a 300% increase of GABAergic input at 1 µM Nic. The drop below baseline in our simulations matches experimental data without further fitting of the model (Fig. 3E).

The present model furthermore accounts for the increase of glutamatergic (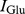) input to the DA cells in response to a 2 min application of 1 µM Nic under GABA block (Fig. 3D). This concentration-dependent increase stems from activation of the presynaptic α7 nAChRs by nicotine (Fig. 3F). The model accounts for a number of experimental data: in control condition (green line and squares), in the presence of an α7 nAChR specific antagonist (orange line and square), and in the presence of an antagonist for non- α7 nAChRs (green line and cyan square).

To account quantitatively for the nicotine-induced increase in Glu input to DA cells (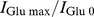) we set the mean firing rate of the Glu afferents (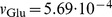) such that the relative increase attains 325% of baseline level. This intermediate value satisfies the experimental data obtained both in the control case (400% increase) and with the α4β2 nAChRs blocked (275% increase, see [19] and Fig. 3F). In the model, blocking the α4β2 nAChRs does not have an effect on the Glu input change since the nicotine-dependent increase stems from activation of α7 nAChRs only (Fig. 3B, D and F). We use the ACh rate as fixed above (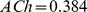 µM) which results in a weak activation of α7 nAChRs on average in the absence of nicotine.

In summary, we show that the model reproduces the relative change in IPSC and EPSC frequency to VTA DA neurons during *in vitro* nicotine perfusions. The model also accounts for the supra-linear (increasing slope) increase in EPSC frequency in the nicotine range from 0.1 to 1 µM Nic (green line and squares in Fig. 3F) and predicts a sub-linear increase (decreasing slope) for the IPSC frequency in the same range of nicotine (Fig. 3E). This difference stems from the dissimilar potencies of Nic for the α7 and the α4β2 receptors: the high Nic potency α4β2 starts to saturate in this range.

### Nicotine-evoked increase of DA cell activity *in vivo*: Direct stimulation vs. disinhibition mechanisms

To translate the VTA circuit model to *in vivo* conditions, we kept all parameters fixed and modified only the afferent input strength. This “increased input” model accounts for the following *in vivo* data : (i) An intravenous injection of nicotine in wild-type mice increases the firing rate of DA cells. (ii) This increase is completely abolished in α4β2 knockout mice and is only weakly diminished in α7 knockout mice [17].

**Figure 4:**
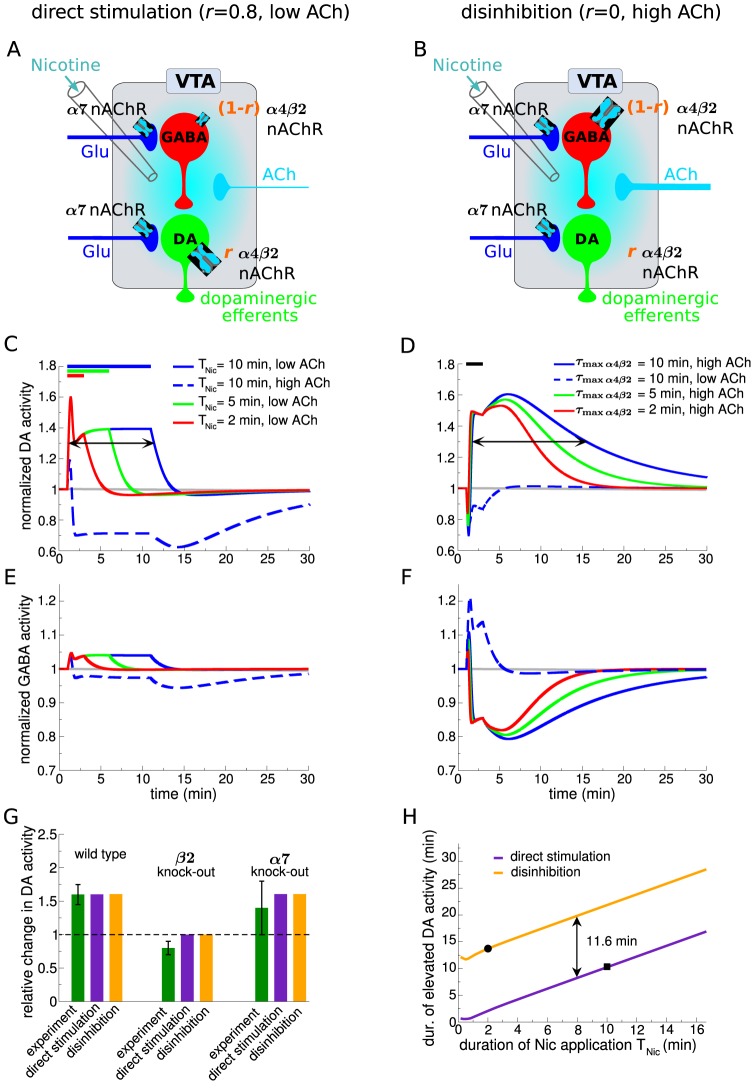
Model VTA responses to nicotine *in vivo*. Panels on the left-hand side (A,C,E) show results of the direct stimulation scenario (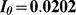 **,** 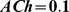 **µM,** 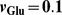) and panels on the right-hand side (B,D,F) depict results for disinhibition (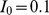 **,** 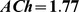 **µM,** 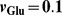 **).** (A&B) Illustration of the simulated experimental situation *in vivo*. Note the difference in α4β2 nAChR distribution between the direct stimulation (A, 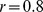) and the disinhibition case (B, 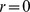). (C&E) Normalized DA (C) and GABA neuron activity (E) in response to the application of 1 µM nicotine in case of direct stimulation. The full lines show the time course of the normalized 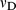 (C) and 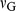 (E) for three different durations of nicotine exposure, 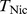 (as indicated by the bar on top of C). The full and the dashed blue lines depict the responses for low (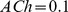 µM) and high endogenous cholinergic input rates (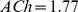 µM), respectively (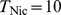 min). (D&F) Normalized DA (D) and GABAergic neuron activity (F) in response to 1 µM nicotine for 2 min in case of disinhibition. The full lines show the time course of the normalized 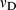 (D) and 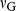 (F) for three different maximal desensitization time constants of α4β2 nAChRs, 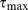 (as indicated in the upper panel). The full and the dashed blue lines depict the responses for high (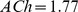 µM) and low cholinergic input rates (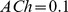 µM), respectively (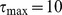 min). (G) Comparison of model results (purple and orange bars) and experimental data (green bars) on relative DA neuron activity changes in response to 1 µM nicotine. The maximal relative increase of DA activity in wild type (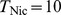 min for direct stimulation; and 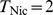 min with 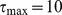 min for disinhibition) and mutant mice is shown (experimental data adapted from [17]). (H) Comparison of the total duration of elevated DA neuron activity with respect to the duration of Nic application, 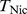. The duration of elevated activity is taken to be the time between the two points where 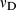 attains half-of-maximum activity (as illustrated by arrows in C and D and depicted by square and circle, respectively). This duration is plotted for direct stimulation (purple) and disinhibition (orange).

We identify two different plausible model regimes that could address the *in vivo* experiments. The scenario in which α4β2 nAChR-mediated action is predominantly exerted through activation on the DA cells is referred to as “direct stimulation” (Fig. 4A). The scenario where α4β2 nAChRs mainly influence the GABAergic activity through desensitization we term “disinhibition” (Fig. 4B). The model can be shifted between these scenarios by a change in the relative expression levels of α4β2 nAChRs on the DA and the GABA neurons using the parameter *r*. To illustrate the qualitative behavior of the model in each scenario, we use 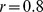 for direct stimulation and we set 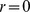 for disinhibition. We do not use the extreme case (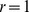) for direct stimulation since the existence of α4β2 nAChRs on GABA cells has been shown experimentally [19], [33]. In general, the conditions for direct stimulation are met as long as 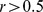 and for disinhibition for 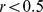 (see below).

Direct Stimulation (single cell level mechanism; 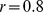) : Our simulations reveal two important requirements necessary for direct stimulation to produce a boost of the DA activity on time scales observed in the data [17]. First, the cholinergic input rate to the VTA has to be low, activating the α4β2 nAChR only weakly (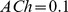 µM; 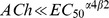 and 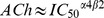). Nic can further activate these nAChRs. Second, we see that the duration of elevated DA activity cannot outlast the presence of Nic (here 1 µM, Fig. 4C), implying that in this scenario the time-course of DA activity is directly determined by the time course of Nic in the VTA.

When the endogenous ACh input rate is increased (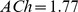 µM), Nic produces only a very brief initial peak followed by a sustained depression of the DA activity (dashed blue line in Fig. 4C). In general we find that as long as more α4β2 nAChRs are expressed on DA than on GABA neurons (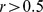), the cholinergic input rate has to be below 0.38 µM in order to observe a net increase of DA activity in response to Nic application (note that we define a DA increase as a positive integral of the difference between the nicotine-modulated- and the baseline DA activity, 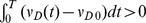).

Disinhibition (circuit level mechanism; 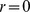) : Our simulations reveal two conditions necessary for the GABA-dependent mechanism of nicotine-evoked DA activity increases. First, we observe that in order for a 2 min exposure to 1 µM Nic to boost DA activity sufficiently to account for the data, the endogenous cholinergic input to the VTA must be high (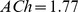 µM, Fig. 4D). Such cholinergic drive assures that nicotine mainly drives α4β2 receptor desensitization after a brief initial period of activation (Fig. 4D,F). Second, the nicotine-induced desensitization suppresses the GABAergic cell response to ACh, reducing it below baseline during and after the exposure to Nic (Fig. 4F, full lines). The return to baseline that follows the Nic removal is governed by the maximal desensitization time constant of α4β2 nAChRs (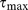; Fig. 4D,F for three different values of 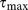). The duration of the DA boost therefore outlasts the presence of Nic as it depends on the recovery of α4β2 nAChRs from desensitization and thereby the removal of inhibition.

When the ACh drive is decreased in the disinhibition case, the DA activity shows a drop below baseline (Fig. 4D, dashed blue line, 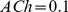 µM), mirroring an increase of GABA cell activity (dashed blue line in Fig. 4F). In general, we find that the endogenous cholinergic input has to be larger than 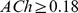 µM in order to see an increase of DA activity in response to Nic.

Our model points out that the two pathways can be disambiguated by the relative dynamical profiles of the DA and GABA neuron responses to nicotine. For the direct stimulation, the activity of the GABAergic neurons (Fig. 4E) matches the DA neuron activity but with a smaller amplitude (due to the smaller fraction of α4β2 nAChRs on GABAergic cells, 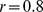). The disinhibition circuit, on the other hand, shows that the profile of DA activity is a mirror-image of the GABAergic activity (compare Fig. 4D and F). For the level of Nic used in our simulations the DA neurons are slaved to the GABA neurons since only the high potency α4β2 receptors on GABA neurons are recruited.

In summary, we demonstrate that direct stimulation, through receptor activation, and disinhibition, through receptor desensitization, require distinct conditions in order to account for nicotine-induced DA activity changes in wild type, α4β2-, and α7-knockout mice (see summary in Fig. 4G). Endogenous cholinergic input rate has to be low for direct stimulation and high for disinhibition. Our simulations further uncover a tell-tale difference in the time course of the DA activity: for direct stimulation the duration of nicotine presence directly determines the duration of the DA response, whereas for disinhibition the recovery from desensitization of the α4β2 nAChRs defines the temporal scale of the DA activity. For a given duration of nicotine application these results predict longer-lasting increases in DA activity with disinhibition as compared to direct stimulation (in our model the difference is on the order of 12 min, Fig. 4H).

### Predictions and experimental protocols to pin down the major pathway of nicotine action

In view of our results, the experimental data available so far are not sufficient to determine conclusively to what extent direct stimulation or disinhibition of DA cells is at the origin of DA responses to Nic. In addition to the differences we uncover above, we propose a series of feasible experiments to conclusively determine whether direct stimulation or dishinibition of DA cells is at the origin of DA responses to Nic. First, we examine the DA cell response for a range of endogenous cholinergic input rates and nicotine concentrations. We then vary the balance of α4β2 nAChRs between DA and GABA cells and investigate the DA response to Nic in the *in vitro* (low endogenous ACh input) and the *in vivo* (high endogenous ACh input) regimes.

**Figure 5:**
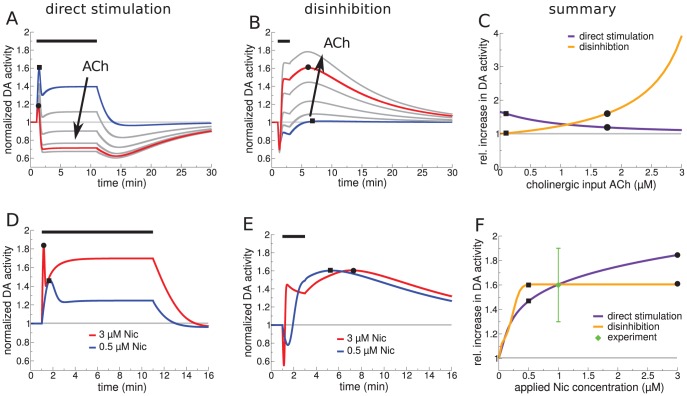
Predicted dynamics of the DA neuron population in the VTA in response to varying cholinergic input rates and Nic concentrations in case of direct stimulation (A,D) and disinhibition (B,E). Nic was applied for 10 min in the direct stimulation scenario, and for 2 min in the disinhibition scenario, whereas the endogenous ACh input rate is modeled to be constant (see text). (A&B) Temporal dynamics of DA neuron activity in response to 1 µM nicotine and varying endogenous cholinergic input rates (ACh range: 0.1-blue lines, 0.5, 1.0, 1.5, 1.77-red lines, 2.0 µM). (C) Maximal increase of DA activity in response to 1.0 µM Nic as a function of the cholinergic input rate (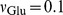, 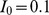). (D&E) Temporal dynamics of DA activity in response to 0.5 µM (blue lines) and 3 µM nicotine (red lines) in case of direct stimulation (D, 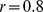, 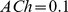 µM, 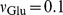, 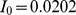), or disinhibition (E, 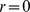, 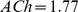 µM, 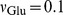, 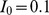). (F) The maximal DA response as a function of applied Nic concentration is depicted for direct stimulation (orange line) and disinhibition (purple line). Data point (green) adapted from [17].

In the first set of experiments we suggest to measure how the DA response to physiologically relevant nicotine injections (*e.g.*, 1 µM Nic) depends on the endogenous ACh input rate. The endogenous ACh rate could be manipulated by stimulation or inhibition of activity in the laterodorsal tegmental nucleus or the pedunculopontine tegemental nucleus (Fig. 1A). We predict that for direct stimulation, the DA response should decrease with rising ACh input rate (Fig. 5A,C). In contrast, disinhibition implies that the total boost in DA activity should increase with the ACh input (Fig. 5B,C). These differences are explained as follows: the ACh drive sets the basal level of activated α4β2 nAChRs and available to be desensitized. At high ACh levels, for direct stimulation, the Nic-induced desensitization of α4β2 nAChRs reduces the excitatory drive to the DA, depressing the DA activity. In contrast, under disinhibition, α4β2 nAChR desensitization decreases the excitatory drive to the GABA cells (Fig. 5B,C), removing the inhibition to the DA population and hence liberating the DA activity. Consequently, the boost of DA activity would become more pronounced under high ACh input rates. The result that the endogenous ACh input rate determines the nAChR-mediated action of Nic holds for a large range of receptor characterizations when actylcholinesterase activity is high (see below and Text S1).

A second set of experiments should examine the dependence of DA activity on the injected Nic dose at a pre-set endogenous ACh input rate. Our model indicates that the two pathways imply distinct time scales for the maximal DA activity. Direct stimulation results in a DA peak directly at the onset of Nic injection (Fig. 5D), whereas the disinhibition case gives a delayed peak after the Nic clearance (Fig. 5E). Moreover, for direct stimulation, the peak amplitude of the maximal DA response increases with rising Nic dose, but levels off rapidly (at ∼0.5 µM nicotine) for the disinhibition case (Fig. 5F). Again, the difference is explained by the dynamics of the α4β2 receptor in response to nicotine. The maximal increase for direct stimulation is due to fast activation of α4β2 nAChRs, whereas in the disinhibition case, the increase arises due to the delayed desensitization of these receptors. For direct stimulation, higher nicotine levels result in stronger receptor activation and an increase in peak amplitude. In contrast, the fraction of receptors driven into the desensitized state saturates at lower Nic for disinhibition with the peak amplitude saturating. This is because the maximal fraction (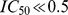 µM) and the minimal rate of desensitization, 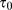, (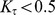 µM) are already attained at ∼0.5 µM nicotine (see Table 1). Note that in these simulations Nic is applied for 2 min in the case of disinhibition and for 10 min in the case of direct stimulation in order to achieve comparable durations of DA activity increases (compare Fig. 4H).

**Figure 6:**
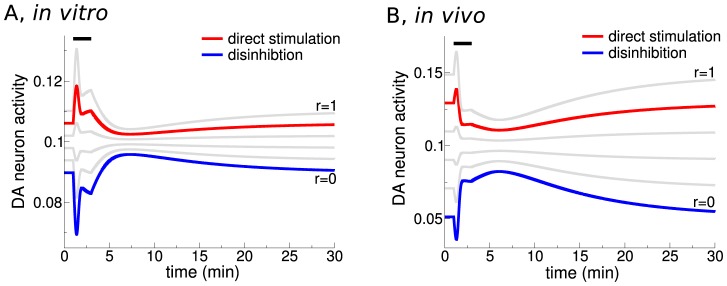
Temporal dynamics of DA neuron activity in response to 1 µM nicotine for 2 min for different values of *r* and afferent input strengths. (A) The DA response in the presence of constant low cholinergic and glutamatergic afferent input to the VTA, *i.e.*, *in vitro* like conditions (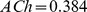 µM,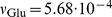). (B) The DA response in the presence of constant high cholinergic and glutamatergic afferent input to the VTA, *i.e.*, *in vivo* like conditions (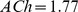 µM, 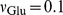). In both panels, the distribution of α4β2 nAChRs is changed by varying the control parameter *r* in steps of 0.2 from 0 to 1 (as indicated). The examples with values of *r* as used for the direct stimulation (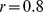, red) and disinhibition (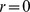, blue) cases in this study are highlighted.

Shifting the balance of the α4β2 nAChR action between the DA and the GABA cells allows to further clarify how the interplay between α4β2 nAChR expression and cholinergic input levels conjointly determine DA activity. In order to do so, we study the acute DA response to 1 µM nicotine for *in vitro* (Fig. 6A) and *in vivo* conditions (Fig. 6B) while varying the value of *r* continuously. For the *in vitro* regime (low afferent input), the net DA activity is increased as long as 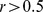 (*e.g.*, direct stimulation case: 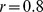, red curve in Fig. 6A). On the other hand, *in vivo* (high afferent input) a net DA increase (after a small negative transient) requires 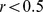 (*e.g.*, disinhibition case: 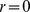, blue curve in Fig. 6B). The extent to which α4β2 nAChR mediated effects inhibit DA cells for low (GABA mediated inhibition for 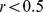, Fig. 6A) and high (desensitization of α4β2-mediated inhibition for 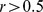, Fig. 6B) endogenous ACh input scenarios depends on the strength of the glutamatergic input to DA cells. In the model, we set the ratio of Glu to GABAergic input weight, 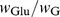, as well as Glu to direct α4β2 nAChR input weight, 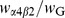, to unity. Had we chosen 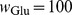, 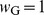 and 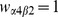, for example, the DA cell activity would exhibit no decrease in activity neither in the *in vitro* nor in the *in vivo* scenarios (results not shown). The case 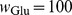, 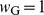 and 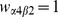 would mean that DA cell activity is dominated by nicotine driven Glu input increases, giving more significance to the α7 nAChRs in generating nicotine-dependent DA responses.

Our analysis reveals key experimental manipulations that would pinpoint the mechanistic basis for nicotine effects in the VTA: manipulating the cholinergic drive to the VTA during nicotine application, and/or changing the nicotine concentration administered. Decreasing the cholinergic drive to the VTA further boosts DA activity increases for direct stimulation but diminishes them for disinhibition (Fig. 5C). The maximal DA response saturates at low nicotine concentrations (∼0.5 µM) for disinhibition, but continues to rise with higher nicotine concentrations for direct stimulation (Fig. 5F).

### ACh-driven desensitization through low acetylcholinesterase activity

In all results reported so far, receptor desensitization is driven by nicotine only. We now investigate what happens if nicotine *and* acetylcholine together drive receptor desensitization of the dominant α4β2 subtype. This might occur whenever acetylcholinesterase activity is reduced. In the model, we vary the amount of ACh contributing to α4β2 nAChR desensitization by the parameter η and study the impact on GABA and DA neuron responses for direct stimulation and disinhibition. η varies between 0 - only Nic drives desensitization - and 1 - Nic and the ACh induce desensitization to equal amounts (see Models).

**Figure 7:**
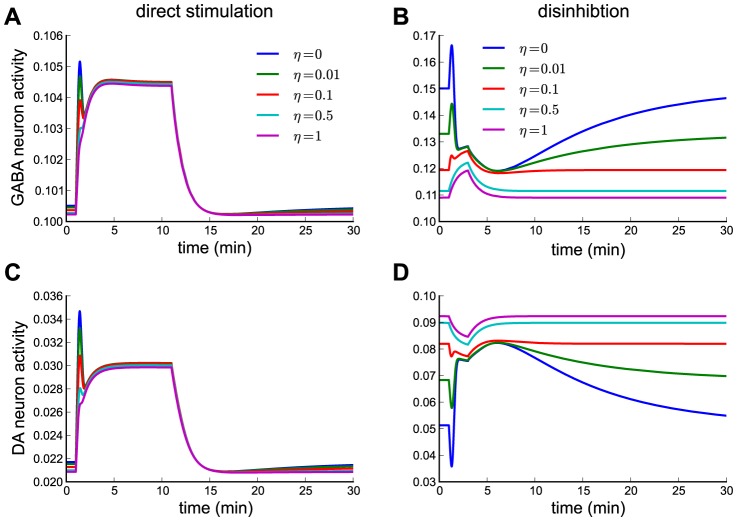
Temporal dynamics of GABA and DA neuron activity in response to 1 µM nicotine in case α4β2 receptor desensitization is driven by Nic *and* ACh. (A,C) GABA (A) and DA (C) neuron activity in the direct stimulation case (same as in Fig. 4C,E full blue lines; 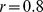, 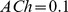 µM, 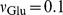, 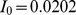) for different efficacies of ACh to drive α4β2 receptor desensitization (η given in panel A). (B,D) GABA (B) and DA (D) neuron activity in the disinhibition case (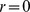, 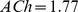 µM, 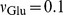, 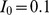) for different efficacies of ACh to drive α4β2 receptor desensitization (η given in panel B, see Models).

Direct stimulation is only marginally affected by ACh-driven desensitization since the α4β2-mediated receptor current predominantly arises from activation through Nic (Fig. 7A,C). The weak endogenous ACh rate induces initial desensitization which reduces the steady-state current but also diminishes the amount of further desensitization through Nic resulting in a negligible net effect (Fig. S1).

In contrast, for the disinhibition scenario, the α4β2 nAChR-mediated current changes from inhibitory to excitatory with increasing the amount of ACh-driven desensitization (Fig. 7B). The high endogenous ACh rate induces strong desensitization and further desensitization by Nic is not enough to overcome receptor activation (Fig. S1E). In turn, GABA neuron activity is increased in response to Nic inhibiting DA neuron activity (purple lines in Fig. 7 B,D).

In summary, acetylcholinesterase activity determines Nic action for disinhibition but has little effect on direct stimulation. In case Nic action is mediated through GABAergic neurons (disinhibtion), varying ACh hydrolysis rates could provide a dynamical means to control Nic action.

## Discussion

The major goal of this study was to determine the dominant pathway of action for nicotine in the ventral tegmental area. In order to do so we have developed a novel mesoscopic computational modeling approach extending a population activity representation of the VTA DA and GABA neurons to describe nAChR responses. This allowed us to clarify the interplay of the pharmacodynamics of nicotine and the dopaminergic signal constructed in the VTA. Our analysis of the model showed that *in vitro* and *in vivo* data can be reconciled by taking into account the difference in the afferent input strengths to the VTA in the two experimental settings: low for *in vitro* and high for *in vivo*. The differential activation and desensitization kinetics of α7- and α4β2 nAChRs combined with different afferent input levels can explain the mechanism of nicotine action.

However, available experimental data have not allowed to pinpoint whether α4β2 nAChRs on VTA DA (direct stimulation) or GABA cells (disinhibition) are the dominant site of nicotine action *in vivo*. Using the model, we demonstrate that both disinhibition and direct stimulation of DA cells can potentially be at the origin of the experimentally observed nicotine-induced boost of DA activity in agreement with the recent suggestion that both receptor activation and desensitization play a role in nicotine influence [34]. Critically we identify that the endogenous cholinergic input rate has to be low for direct stimulation, whereas high cholinergic inputs are crucial in the disinhibition case, in order to observe a DA activity increase. These results emerge directly from known activation and desensitization properties of α4β2 nAChRs.

Several experimental results support the critical role of the GABAergic cells for the action of nicotine. Most of the cholinergic axon terminals in the VTA synapse on the non-DA neurons [35]. The α4-containing nAChRs expressed on the VTA GABAergic neurons upregulate in response to chronic nicotine, potentially boosting the nicotine control of their activity [28], [36]. The activation of VTA GABA neurons is necessary for the reinforcing actions of Nic [37]. A wide class of addictive drugs, including opioids, cannabinoids, γ-hydroxy butyrate (GHB), benzodiazepines, lead to inhibition of GABA neurons in the VTA and thereby disinhibition of DA neurons (*e.g*, [38], see [39] for an overview). Furthermore, the GABA antagonist bicuculline is self-administered by mice [40], [41]. Taken together these facts support the hypothesis that reduced GABAergic input to DA cells can initiate addictive behaviors. The hyperexcitability of the VTA in response to nicotine [42] could be related to the higher abundance of GABAergic cells in the VTA as compared to the SN (GABA to DA ratio about 1/4 in the VTA [43]; and 1/19 in the substantia nigra [44]). We would like to point out that the disinhibition scenario emphasizes the role of local circuitry organization as opposed to the single cell mechanism associated with direct stimulation of DA cells. It may also be that disinhibtion is primarily due to nicotine-induced inhibition of GABA activity of extrinisic rather than local GABA neurons. Candidates include GABAergic projections onto DA neurons that arise in the ventral palladium or the rostromedial tegmental nucleus [45], [46].

Our model makes several testable predictions for the case that the reinforcing properties of nicotine are mediated by inhibition of the GABA cells. Notably, the DA activity response to Nic is a mirror-image of the GABAergic firing response (Fig. 4D,F). Moreover, the DA response to Nic is biphasic: the longer boost is preceded by a short-lasting inhibtion due to the fast activation of α4β2 nAChRs (Fig. 4D, and 5B). Some DA neuron recordings appear to support this observation [47]. We further predict that the DA boost should saturate at relatively low Nic levels (∼500 nM) in case of disinhibition (Fig. 5F). Higher nicotine levels do not evoke further increases of DA activity since the maximal desensitization of α4β2 nAChR is already attained at low nicotine. This result implies that Nic elicits maximal α4β2 nAChR-mediated increase of DA activity at nicotine concentrations attained in the blood of smokers [11], [31].

We confirm previous findings suggesting that physiologically relevant doses of nicotine do not significantly desensitize α7-containing nACh receptors [48]. We extend this statement and propose that physiological concentrations of nicotine do not significantly activate α7-containing nAChRs. We consider it therefore unlikely that increased glutamatergic drive to DA cells in response to nicotine augments their activity. It should however be noted that the mean-field approach presented here does not resolve the different firing modes of DA cells, *i.e.*, bursting and regular firing. α7-containing nAChs could play a role in nicotine induced bursting [49], [50]. The tonic inhibitory input from GABAergic cells may be setting the overall level of excitability of DA cells by controling the mean membrane potential. When the DA cells are disinhibited through desensitization of α4β2 nAChRs, the α7 nAChR activation could induce burst firing on top of the elevated membrane potential. Hence GABA cells would gate the DA burst firing, as suggested in a biophysically detailed model by Komendantov et al. [51]. Furthermore, bursting induced by α7 nAChR activation could be crucial for the induction of long-term potentiation of Glu synapses onto DA cells [18], [52]. These topics remain subject of active investigations.

We note that we focus only on feedforward afferent input (glutamatergic and cholinergic) and a simplified local circuitry of the VTA. While we leave aside the possible involvement of other neuronal structures [46], [53], we find that our setup is sufficient to account for a wide range of data on nicotine/DA interactions. Furthermore, we chose to not address the potential heterogeneity of the VTA itself [35], [54], [55]. However, our proposed circuitry can be seen either as a global description of the VTA or as a model of a local computational unit within the VTA. Whether the experimentally observed diversity of DA cell behavior could be explained by the coexistent presence of direct stimulation and disinhibition subcircuits or whether recurrent inhibition has to be taken into account remains an area for future studies. However, already in our model the activities of the DA and GABAergic cells show a variety of temporal profiles depending on the α4β2 nAChR expression and the cholinergic input rate.

Data and theory suggest that dopamine levels modulate synaptic plasticity and learning [56], [57]. Our results together with this fact lead us to speculate that if salient characteristics of environmental cues are reflected in the overall cholinergic tone [58], the nicotine induced increase of phasic DA may explain the strong associations formed between these cues and the habit of smoking [59], [60].

Our results provide a clear paradigm for understanding the interactions between endogenous acetylcholine input and the mechanism by which exogenous nicotine may provoke DA changes: differential control over the local VTA mechanisms by endogenous ACh input rates. Endogenous ACh inputs determine whether Nic evokes a positive DA response or a DA depression for either scenarios discussed here, *i.e.*, direct stimulation and disinhibition. For example, nicotine-evoked DA increases and high ACh input imply disinhibition, while DA increases and low ACh implicate direct stimulation. Furthermore, taking diural rhythms of cholinergic signaling into account, the VTA may give rise to different DA outputs at different times of the day, *e.g.*, the morning cigarette may deploy different mechanisms than an evening cigarette. In turn this state-dependency of nicotine-induced reinforcement could imply that the therapeutic strategies for smoking cessation may need to be tailored for a variety of cognitive states in order to act on specific targets within the predominant pathway of nicotine action.

## Models

In order to examine the mechanisms of nicotine action, we built a neural population model of the ventral tegmental area microcircuit using the mean-field approach [61]. Our minimal local circuit model of the VTA incorporates the glutamatergic (Glu) and cholinergic (ACh) afferents to the DA and GABA cells in the VTA, as well as local inhibition of DA cells by GABA neurons (Fig. 1A). The activation and desensitization of the nAChRs in response to Nic and ACh were described by a simple 4-state model adapted from [22], [23] (Fig. 1B,C; see Text S2.).

### Mean-field description of dopaminergic and GABAergic VTA neurons

The temporal dynamics of the average activities of dopaminergic and GABAergic neuron populations is characterized by
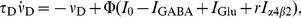
(1)

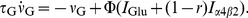
(2)

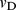
 and 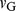 are the mean firing rates of the DA and GABAergic neuron populations, respectively. 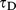 and 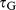 are membrane time constants of the neurons specifying how quickly the neurons integrate input changes (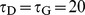 ms). 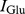 and 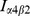 characterize excitatory inputs to both neuron populations mediated by glutamate receptors and α4β2-containing nAChRs, respectively. 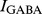 is the local inhibitory input to DA neurons emanating from VTA GABAergic neurons. 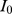 is an intrinsic current of DA cells giving rise to intrinsic activity in the absence of external inputs [62]. We further assumed that 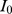 accounts for other input sources which are not affected by nicotine and therefore provide a constant background input (*e.g.*, α6-containing nAChR mediated cholinergic input to DA cells [63]; inhibitory input originating in other brain regions, *etc.*). 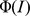 is the steady-state current-to-rate transfer function. For simplicity, we assumed that Φ(*I*) is threshold-linear: 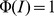 if 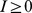 and 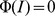 otherwise. The control parameter *r* sets the balance of α4β2 nAChR action through GABAergic or DA cells in the VTA. For 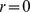: α4β2 containing nAChRs act through GABAergic neurons only, whereas for 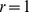: α4β2 receptors influence DA neurons only. Both neuron populations are influenced by α4β2 nAChRs for intermediate values of *r*. In practice, this balance is determined by the expression level of α4β2 nAChRs, the overall impact of local GABAergic inputs on DA activity, and by the location of α4β2 nAChRs on the somatodendritic tree of DA and GABAergic cells.

The input currents in Eqs. (1) and (2) are given by
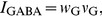
(3)

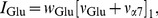
(4)

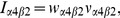
(5)where the 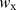
's (with x = G, Glu, α4β2) specify the total strength of the respective input since the activation variables (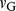, 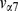, 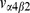) are normalized to vary between 0 and 1. For our qualitative investigations, we used 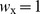 (with x = G, Glu, α4β2). Inhibitory input to DA cells, 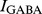, depends on the GABAergic neuron population activity, 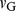. Glutamatergic input is provided either by upstream glutamatergic activity, 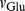, or by activation of α7 nAChRs on presynaptic glutamatergic terminals, 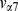 (see below). Nicotine-evoked glutamatergic transmission is independent of action potential activation in presynaptic neurons [18]. Hence, either of both inputs can fully activate glutamatergic transmission
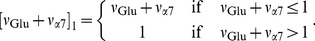
(6)The activation of α4β2 nAChRs, 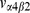 (see next section), determines the level of direct excitatory input, 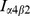, evoked by nicotine or acetylcholine [63].

### Modeling the activation and desensitization of nAChRs driven by Nic and ACh

We implemented nAChR activation and desensitization as transitions of two independent state variables: an activation gate and a desensitization gate. This yields four different states of the nAChR: deactivated/sensitized (also resting or responsive state), activated/sensitized, activated/desensitized and deactivated/desensitized state (Fig. 1B). Of those states, three are closed and the activated/sensitized state is the only open state of the receptor in which it mediates an excitatory current. Note that compared to other models of allosteric transitions of the nAChR, we chose to leave aside the rapidly and slowly desensitized states [64], deeper-level desensitized state or inactivated states [65]. Such states are collapsed in the desensitized state here. Our model was modified from the cyclic desensitization model of Katz and Thesleff [22] where “effective” and “refractory” in their model refer to sensitized and desensitized here, respectively (see Text S2.). Assuming independent transitions of the activation and the desensitization variables entails another simplification compared to cyclic allosteric transition schemes. In our model, the reaction rates are the same on opposite sides of the reaction cycle (Fig. 1B), *e.g.*, the rate from deactivated/sensitized to activated/sensitized is the same as the transition rate from deactivated/desensitized to activated/desensitized.

The model accounts for the opening of the channel (transition from deactivated/sensitive to activated/sensitive, Fig. 1B,C) in response to both Nic and ACh; while desensitization is driven by nicotine and ACh if 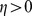 (transition into the activated/desensitized state, Fig. 1B,C). The inverse transitions, *i.e.*, from activated to deactivated and from desensitized to sensitized, occur after the removal of Nic and ACh.

The mean total activation level of nAChRs (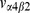, 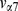) is modeled as the product of the fraction of receptors in the activated state, *a*, and the fraction of receptors in the sensitized state, *s*. The total normalized nAChR activation is therefore 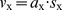 with x = α4β2 or α7. The time course of the activation and the sensitization variables is given by
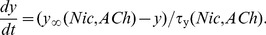
(7)where 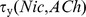 refers to the Nic/ACh concentration-dependent time constant at which the asymptotically achievable steady-state 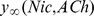 is attained. The maximal achievable activation or sensitization, for a given Nic/ACh concentration, 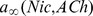 or 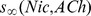 respectively, are given by Hill equations of the form
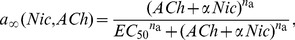
(8)

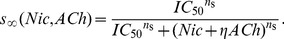
(9)

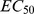
 and 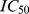 are the half-maximal concentrations of nAChR activation and sensitization, respectively. The factor α>1 accounts for the higher potency of Nic to evoke a response as compared to ACh: 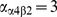, 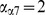 [26]–[28], [30] (see Fig. 2C,D and Table 1). 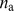 and 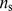 are the Hill coefficients of activation and sensitization. η varies between 0 and 1 and controls the fraction of the ACh concentration driving receptor desensitization.

The transition from the deactivated to the activated state is fast (∼µs, ms) [64] compared to the time scales investigated here that are of the order of seconds to minutes. We therefore simplified the activation time constant, 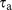, to be independent of the acetylcholine/nicotine concentration, *i.e.*, 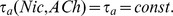. The time course of Nic-driven desensitization is characterized by a concentration-dependent time constant
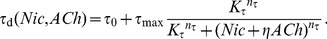
(10)

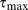
 refers to the recovery time constant from desensitization in the absence of ligands (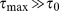). 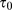 is the fastest time constant at which the receptor is driven into the desensitized state at high ligand concentrations. 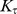 is the concentration at which the desensitization time constant attains half of its minimum (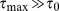). η varies between 0 and 1 and controls the fraction of the ACh concentration influencing the desensitization time constant. The parameters describing activation and desensitization of the two nAChR subtypes were taken from a number of studies on heterologously expressed human nAChRs and are listed in Table 1 [25]–[30]. Note that we used 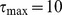 min for α4β2 nAChRs in order to match the time course of DA activity recorded *in vivo*, while Fenster et al. [25] recorded a value of 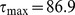 min *in vitro* during experiments at room temperature. We adjusted the minimal time constant by which the receptors are driven into the desensitized state, 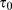, such that the model currents qualitatively captured the experimentally measured time course of nAChR currents evoked by Nic and ACh [25]–[30]. This fit yielded a faster minimal desensitization time constant for the α7 nAChR, *i.e.*, 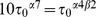 (Table 1).

In our simulations, we assumed that both the nicotine bath application and the intravenous injection imply a slow build-up of the nicotine concentration at the site of the receptor. That is, the applied nicotine concentration is not immediately available but increases/decays exponentially with a time constant of 1 min. The fast activation of nAChRs, *a* (transition from deactivated/sensitive to activated/sensitive, Fig. 1B), was therefore taken to be in steady-state with the Nic concentration at all times (except in Fig. 2).

Clearly, the above presented simple model of nAChR activation and desensitization did not resolve all the details of nAChR kinetics. For example, it was assumed that ACh- and Nic-evoked responses reach the same maximal amplitude and that, despite different potencies, Nic and ACh dose-response curves can be characterized by the same Hill coefficient. These assumptions are approximately met for α7-containing nAChRs [66]. ACh evokes however twice the response of Nic with human α4β2 nAChRs in a study by Chavez-Noriega et al. [66], but the same response according to other studies [28], [67]. We simplified the dose-response curve using a single Hill equation, rather than using a sum of two Hill equations as suggested by Buisson and Bertrand [28]. Nevertheless the simple model presented here captured the qualitative time course of nAChR currents evoked in response to Nic and ACh exposures (see Results, [24]). It furthermore quantitatively accounts for the time course of DA and GABA neuron activity responses *in vivo* for single and repetitive Nic applications [37].

## Supporting Information

Figure S1
**α4β2 nAChR-mediated current for various endogenous ACh input rates and ACh-driven desensitization levels.** Three different levels of ACh-driven desensitization are considered in the three columns (see top of each column; see also Model and Methods). (A,C,E) Steady-state activation (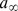, blue) and sensitization (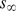, green) curves of the α4β2 nAChR model (Table 1). The three endogenous ACh input rate cases depicted in each column are indicated by arrows. (B,D,F, top) The dynamics of the activation, 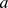, (full lines) and sensitization, 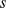, (dashed lines) variables in response to 1 µM nicotine for 2 min. (B,D,F, bottom) The dynamics of the total normalized receptor current during the nicotine application. The total current is given by 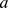 times 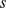. The three color in (B,D,F) correspond to different endogenous ACh input rates indicated in A, C, and E in the same color (A: 0.1, 1.77, 10 µM; B: 0.1, 1.77, 20 µM; C: 0.1, 1.77, 20 µM).(EPS)

Figure S2
**nAChR mediated current in case of no overlap between the activation and sensitization functions.** Same format as in Fig. S1, the mediated current is studied for various endogenous ACh input rates (shown in cyan, magenta and gray; see arrows in A,C,E) and three ACh-driven desensitization levels (see top of each column). (A,C,E) Steady-state activation (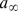, blue) and sensitization (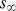, green) curves. The three endogenous ACh input rate cases depicted in each column are indicated by arrows. (B,D,F, top) The dynamics of the activation, 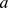, (full lines) and sensitization, 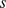, (dashed lines) variables in response to 1 µM nicotine for 2 min. Modified parameters are 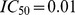 µM and 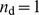, all other parameters are unchanged from the α4β2 nAChR model (Table 1).(EPS)

Figure S3
**nAChR mediated current in case of a large overlap between the activation and sensitization functions.** Same format as in Fig. S1, the mediated current is studied for various endogenous ACh input rates (shown in cyan, magenta and gray; see arrows in A,C,E) and three ACh-driven desensitization levels (see top of each column). (A,C,E) Steady-state activation (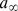, blue) and sensitization (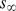, green) curves. The three endogenous ACh input rate cases depicted in each column are indicated by arrows. (B,D,F, top) The dynamics of the activation, 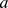, (full lines) and sensitization, 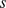, (dashed lines) variables in response to 1 µM nicotine for 2 min. Modified parameters are 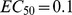 µM, 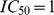 µM and 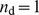, all other parameters are unchanged from the α4β2 nAChR model (Table 1).(EPS)

Figure S4 **The different stages in the construction of the two-gate model for nAChRs. (A) The cyclic model by Katz and Thesleff (1957) [22].** (B) The two-gate model mapped onto the Katz-Thesleff model. (C) Separation of the activation and desensitization gates in the two-gate model. (D) Generic two-gate model with concentration-dependent rate constants. (E) Steady-state curves (*∞*) and (F) respective time-constants (τ) for the activation (*a*, black curves) and sensitization gates (*s*, grey) of the α7 nAChR. See text for details.
(EPS)

Figure S5 **Comparison of the responses generated by the Katz-Thesleff model and the present two-gate model for α7 nAChRs.** (A) Implementation of the Katz-Thesleff model following Papke (2010) (see reference list in Text S2). AR* is the only open state. The rate-constants were hand-tuned to make the model fit the response trace of the two-gate model during the application of a 1 s 100 µM ACh pulse (left-lower panel in B). (B) Response traces of the two-gate model (black curves) and the Katz-Thesleff model (grey) to varying ACh concentrations. The indicated concentrations are those used for ACh in the simulation of the two-gate model. These concentrations were adapted for the Katz-Thesleff simulations to account for the Hill exponent of two in the two-gate model, yielding values for [A] of 1.86, 12.5, 100 and 669 µM, respectively. The open-channel probabilities, plotted on the vertical axes, were calculated as [AR*] for the Katz-Thesleff model, and as *a* times *s* for the two-gate model.
(EPS)

Text S1 **Receptor currents for different activation/sensitization realizations.** We investigate in more detail the α4β2 nAChR mediated current for different levels of ACh-driven desensitization and varying endogenous ACh input rates. We furthermore study receptor implementations with qualitatively different concentration-response profiles for activation and sensitization. These different realizations can be seen as characterizing other subtypes of nAChRs for which we identify excitatory and/or inhibitory receptor current regimes with respect to the ACh input rate and ACh-driven desensitization levels.
(DOCX)

Text S2 **From the cyclic Katz-Thesleff model to the two-gate model.** We explain in greater detail the relationship between the present two-gate model for nAChRs, and the cyclic Katz-Thesleff model from which it was derived.
(DOCX)
