# Supplementary material for: Endogenous Cholinergic Inputs and Local Circuit Mechanisms Govern the Phasic Mesolimbic Dopamine Response to Nicotine
Source: PLoS Comput Biol. 2013 Aug 15;9(8):e1003183. doi: 10.1371/journal.pcbi.1003183 (PMC3744411; doi:10.1371/journal.pcbi.1003183)
Supplement: Text S1 — Receptor currents for different activation/sensitization realizations. We investigate in more detail the α4β2 nAChR mediated current for different levels of ACh-driven desensitization and varying endogenous ACh input rates. We furthermore study receptor implementations with qualitatively different concentration-response profiles for activation and sensitization. These different realizations can be seen as characterizing other subtypes of nAChRs for which we identify excitatory and/or inhibitory receptor current regimes with respect to the ACh input rate and ACh-driven desensitization levels. (DOCX) [file pcbi.1003183.s006.docx]

## Text S1. Receptor currents for different activation/sensitization realizations

## We here investigate in more detail the α4β2 nAChR mediated current for different levels of ACh-driven desensitization and varying endogenous ACh input rates. We furthermore study receptor implementations with qualitatively different concentration-response profiles for activation and sensitization. These different realizations can be seen as characterizing other subtypes of nAChRs for which we identify excitatory and/or inhibitory receptor current regimes with respect to the ACh input rate and ACh-driven desensitization levels.

When increasing the endogenous ACh rate, the effect of the α4β2 nAChR-mediated current changes from excitatory (increase firing), at low ACh rate, to inhibitory (decrease firing), at high ACh rate (Fig. S1A,B). These two different regimes are at the origin of the direct stimulation (low ACh rate) and disinhibtion (high ACh rate) scenarios.

What is the effect of the receptor current in case of impaired acetylcholinesterase activity, *i.e.*, when not only Nic but also ACh drives desensitization. The dynamics of the α4β2 nAChR-mediated current is increasingly dominated by Nic-induced receptor activation which results in an excitatory net current (Fig. S1D,F). The level of ACh input rate determines the initial fraction of desensitized and activated receptor (for η>1). Strong initial activation through ACh input also implies strong initial desensitization for the α4β2 nAChR (see gray lines in Fig. S1F). In turn, further Nic-induced activation and desensitization are small, leading to a weak inhibitory current at low ACh-driven desensitization levels (η=0.1, gray lines in Fig. S1 D), and a weak excitatory current at high ACh-driven desensitization levels ($\eta=1$, gray lines in Fig. S1F).

We next investigate nAChR-mediated currents for receptor models with different parameter sets for the steady-state activation and sensitization functions ($a_{\infty}$and $s_{\infty}$) compared to the α4β2 nAChR implementation. In particular, we consider two qualitatively different cases : (i) the asymptotic activation and sensitization functions show little overlap (Fig. S2), and (ii) the asymptotic activation and sensitization functions show a large overlap giving rise to a large window current (Fig. S3). We ask whether nAChRs with such activation/sensitization characteristics mediate excitatory and/or inhibitory currents.

All three considered nAChR model implementations (Fig. S1, S2, S3) share the same behavior when Nic alone drives desensitization: Nic elicits an excitatory current for low ACh input rates which turns into an inhibitory current at high ACh input rates (note that the specific ACh levels vary). Here, activation and desensitization are driven independently by the ACh input rate and by the Nic concentration, respectively. It is therefore always possible to find a ACh level which induces little activation such that Nic action is dominated by further receptor activation (cyan lines in Fig. S1B, S2B, S3B). Alternatively, high ACh levels (with respect to the $a_{\infty}$ curve half-activation) impair further activation by Nic and Nic action is dominated by desensitization (gray lines in Fig. S1B, S2B, S3B).

When ACh *and* Nic drive desensitization, activation and desensitization are linked and cannot be controlled independently. Whether Nic elicits an excitatory or inhibitory current is therefore determined by the relative position between activation and sensitization curves. When activation and desensitization show no or little overlap, as it is the case for the α4β2 nAChR ($IC_{50}<EC_{50}$; examples in Fig. S1 and S2), Nic elicits an excitatory current since the extra desensitization through Nic is not enough to counterbalance activation and turn the total current inhibitory (Fig. S1F, S2F). In contrast, in case of a large overlap between activation and sensitization ($IC_{50}>EC_{50}$, example in Fig. S3), Nic can elicit an inhibitory current since appropriately chosen ACh ($ACh>EC_{50}$) input rates activate but barely desensitize the receptor and Nic predominantly desensitizes the receptor (gray line in Fig. S3). This case is equivalent to the inhibitory current elicited by Nic when only Nic drives desensitization together with high ACh input rate (gray lines in Fig. S1B and Fig. S2B).

In summary, our conclusion that the endogenous ACh input rate determines the nAChR mediated action of Nic is true for a variety of receptor characterizations when the acetylcholinesterase activity is high (Nic-driven desensitization), or when receptor activation occurs at lower ligand concentrations than desensitization (see Fig. S3). When acetylcholinesterase activity is reduced, Nic-evoked excitatory currents dominate if activation occurs at equal or higher ligand concentrations compared to desensitization.
