# Supplementary material for: Endogenous Cholinergic Inputs and Local Circuit Mechanisms Govern the Phasic Mesolimbic Dopamine Response to Nicotine
Source: PLoS Comput Biol. 2013 Aug 15;9(8):e1003183. doi: 10.1371/journal.pcbi.1003183 (PMC3744411; doi:10.1371/journal.pcbi.1003183)
Supplement: Text S2 — From the cyclic Katz-Thesleff model to the two-gate model. We explain in greater detail the relationship between the present two-gate model for nAChRs, and the cyclic Katz-Thesleff model from which it was derived. (DOCX) [file pcbi.1003183.s007.docx]

## Text S2. From the cyclic Katz-Thesleff model to the two-gate model

We here explain in greater detail the relationship between the present two-gate model for nAChRs, and the cyclic Katz-Thesleff model from which it was derived.

Our reasons for simplifying the Katz-Thesleff model were twofold: computational efficiency, especially when the channels are used as components of neuronal circuit models, and an enhanced flexibility in the assessment of kinetic parameters from available experimental data.

In its original notation, the cyclic Katz-Thesleff ([Katz & Thesleff, 1957](#_ENREF_3)) model (Fig. S4A) describes the kinetics of receptor channels that are either receptive (R) or desensitized (D), and bound to agonist A or unbound. Only in the AR state is the channel conducting. Eight rate constants govern the transitions between states. Typically, agonist binding is fast whereas de- and resensitization are slow. Although the complete set of rate constants can be determined experimentally from the statistics of single-channel opening, this has only been done for particular receptor subtypes and particular agonists (see for instance ([Auerbach & Akk, 1998](#_ENREF_1); [Demazumder & Dilger, 2001](#_ENREF_2))).

The cyclic Katz-Thesleff model can be made symmetrical (Fig. S4B) if both the activation and desensitization are agonist-driven and if the paired rate-constants of transitions in the same direction are identical ($k_{i}=k_{i}^{'}$, $i=1...4$). This symmetrical model then formally separates into two gates, *a* and *s*, with independent kinetics (Fig. S4C). The values $a^{1}$ and $s^{1}$ indicate the fractions of channels in the active and sensitive states, respectively. The product of $a^{1}$ and $s^{1}$ yields the fraction of open channels.

The linear dependence of the forward-reaction rates on agonist concentration [A] (as in Fig. S4C) can be generalized to arbitrary dependencies by using rate-constants that are themselves functions of [A] (Fig. S4D). At each given concentration [A], the activation kinetics is first-order linear with equilibrium $y_{\infty}(A)=k_{1}(A)/(k_{1}(A)+k_{2}(A))$ and time-constant $\tau_{y}(A)=1/(k_{1}(A)+k_{2}(A))$, and likewise for the *s* gate. The equilibrium values and time-constants can be Hill equations fitted to dose-response curves, or be provided as tables. In this form, the two-gate model becomes equivalent to the Hodgkin-Huxley model for voltage-gated ion channels. Figure S4E and F depict the steady-state and time-constant functions used for the activation and sensitization gates of the a7 nAChR.

To compare the kinetics of the present two-gate model with that of the original Katz-Thesleff model, we first re-tuned the rate constants of the Katz-Thesleff model implemented by ([Papke, 2010](#_ENREF_4)) (depicted in Fig. S5A), so as to fit the response of the two-gate model to a step application of 100 μM ACh (see Fig. S5B, left lower panel). We then compared, using the same parameter values, the response traces to other agonist concentrations (other panels in Fig. S5B). Residual differences between the traces, especially in the rate of desensitization, were due to cooperation in the sentization gate of the two-gate model (Hill coefficient of 2), not accounted for in the modeled Katz-Thesleff kinetic scheme.

To conclude, the two-gate model is generic and flexible because its parameters can take the form of dose-response curves, which is the format experimental data are most often presented in. At the same time, it retains most of the kinetics of the Katz-Thesleff model, such as the concentration-dependent transition rate enhancement of activation and desensitization, the stronger binding of agonist (higher affinity) to receptors in the desensitized state, and the preferential desensitization of receptors from the open state. The latter characteristic is a consequence of the fact that the two gates, although formally independent, are anticorrelated by the shapes of their dose-response functions.

**References**

Auerbach, A, & Akk, G. (1998). Desensitization of mouse nicotinic acetylcholine receptor channels. A two-gate mechanism. *J Gen Physiol, 112*, 181-197.

Demazumder, D., & Dilger, J. P. (2001). The kinetics of competitive antagonism by cisatracurium of embryonic and adult nicotinic acetylcholine receptors. *Mol Pharmacol, 60*(4), 797-807.

Katz, B., & Thesleff, S. (1957). A study of the desensitization produced by acetylcholine at the motor end-plate. *J Physiol, 138*(1), 63-80.

Papke, R. L. (2010). Tricks of perspective: insights and limitations to the study of macroscopic currents for the analysis of nAChR activation and desensitization. *J Mol Neurosci, 40*(1-2), 77-86. doi: 10.1007/s12031-009-9261-0
